# Content Analysis of Electronic Nicotine Delivery System Publications in Core Clinical Journals from 2012 to 2018

**DOI:** 10.3390/ijerph17072201

**Published:** 2020-03-25

**Authors:** Michael Briganti, Olivia A. Wackowski, Cristine D. Delnevo, Leanne Brown, Shirin E. Hastings, Binu Singh, Michael B. Steinberg

**Affiliations:** 1 Rutgers Center for Tobacco Studies, New Brunswick, NJ 08901, USA; mb1629@sph.rutgers.edu (M.B.); wackowol@sph.rutgers.edu (O.A.W.); delnevo@sph.rutgers.edu (C.D.D.); bs649@sph.rutgers.edu (B.S.); 2 Department of Medicine, Rutgers Robert Wood Johnson Medical School, New Brunswick, NJ 08901, USA; leannemb@rwjms.rutgers.edu (L.B.); hastinse@rwjms.rutgers.edu (S.E.H.)

**Keywords:** electronic cigarette, e-cigarette, electronic nicotine delivery system, publications, physician, medical journal, clinical journal

## Abstract

There is no consensus if electronic nicotine delivery systems (ENDS) should be used to reduce harm among the smoking population. Physicians, who represent a trusted source of health communication, are exposed to a range of often conflicting ENDS information and this information may be relayed to patients looking to quit smoking. Previous studies have examined ENDS content of various sources of media but there is a lack of knowledge about ENDS content in medical journals. We analyzed 421 ENDS publications printed between 2012 and 2018 from PubMed’s Core Clinical Journal list. Publications were analyzed based on publication type, journal type, study design, geographic focus, theme, terminology, outcomes, and positive/negative statements. The number of ENDS publications grew since 2012, and peaked in 2015. Across all years, negative statements about ENDS outnumbered positive statements, though this difference decreased over time. Over time, negative statements about “toxins and carcinogens” were made less frequently, while negative statements about “gateway effects” and “youth appeal” became more prevalent. UK journals had fewer negative statements relative to US journals. Only 12.6% of publications included guidance for healthcare professionals about ENDS. As published ENDS topics change over time, physicians’ communications with patients may be impacted.

## 1. Introduction

Electronic nicotine delivery systems (ENDS) are battery-powered devices that deliver nicotine and flavors to a user through an inhaled aerosol generated by vaporizing a heated liquid. ENDS have been available in the United States since 2006 [1], and became a common product in traditional retail environments in 2010 [2]. ENDS represent a heterogeneous group of products that come in different styles, and a users’ exposure to toxicant and nicotine levels can depend on the type of device and how the device is used [3]. Among adults, ENDS are predominantly used by smokers for smoking cessation, smoking reduction, and harm reduction [4]. Smokers also find ENDS more appealing than traditional nicotine replacement therapy products [5,6]. 

There is no consensus among healthcare providers regarding recommending ENDS to patients who smoke as a means to reduce harm. Since ENDS contain lower levels of toxicants compared to combustible tobacco [7], some believe it is reasonable for physicians to support using ENDS as a tobacco cessation aid [8]. Indeed, between 2016–2018, influential organizations in multiple countries, such as the Royal College of Physicians in the United Kingdom [9], the National Academies of Sciences, Engineering and Medicine [10], and the American Cancer Society in the United States [11], have suggested that ENDS could pose reduced harm and possibly a cessation benefit. Regardless, research studies and media headlines have voiced persisting concerns that ENDS contain toxicants and pose health risks, that the long term-effects of ENDS are unknown, that ENDS may facilitate dual use with smoking rather than complete switching, and that their availability and promotion are leading to new use and addiction among youth. As a result, patients and physicians are exposed to conflicting information about ENDS.

Physicians are an important and trusted source of health information for patients [12,13]. Several studies show patients ask their physicians about ENDS and some physicians recommend them to patients [14,15], a finding more likely among physicians who believe ENDS to be less harmful than smoking [16]. Physicians’ knowledge and attitudes about ENDS may be influenced by the sources of ENDS information they are exposed to. Among a focus group of Swiss physicians, literature and the internet (which included online literature) were identified as important sources physicians used to inform their knowledge [17]. The medical professional network Doximity, of which over 70% of US physicians belong to [18], found 98% of physicians report literature impacts clinical practice and 75% of physicians change clinical practice quarterly or monthly as a result of medical literature [19]. 

Journals are one of many sources of information physicians use to inform practice decisions and it is important to understand the e-cigarette content and messages of these sources, particularly with regard to the safety and effectiveness of ENDS. Several studies have analyzed the content of ENDS material in the news [20], magazines [21], social media [22], and websites [23]. However, there is a lack of information about ENDS scientific publications in medical journals. The purpose of this study is to examine the frequency of ENDS publications, trends in ENDS terminology used, and topics/themes covered, and ENDS risk and benefit statements included in medical journals over time. 

## 2. Materials and Methods 

This study utilized searches in PubMed, an online citation database developed by the National Center for Biotechnology Information which includes access to over 26 million scientific articles [24]. Electronic cigarette publications were queried that physicians are likely to be exposed to by limiting our search to articles categorized under PubMed’s “Core Clinical Journals”. This subset currently includes 118 journals [25]. The journals on the Core Clinical list are selected by a committee whose purpose is to review and update the list over time, as well as from survey data collected from hospital libraries which gather the most requested journals from physicians [26]. 

The Core Clinical list was searched for all documents published between 2012 and the end of 2018. Publications were included if they contained at least one of the following keywords under ‘All Fields’: “electronic cigarette(s)”, “electronic nicotine delivery system(s)”, “e-cig(s)”, “e-cigarette(s)”, “vape”, “vaping”, and “JUUL”. JUUL was the only brand name searched for due to its dominance of the e-cigarette market share [27]. Authors with the name Juul were excluded. 

Publication types were categorized as defined by Scopus [28] and included “Article”, “Review”, “Note”, “Letter”, “Editorial”, “Short Survey”, and “Undefined”. Scopus defines a short survey as a miniature review with a short bibliography. We excluded “Erratum”, “Book Chapter”, “Conference Review”, and “Book” from analysis. “Erratum” and “Conference Reviews” were excluded to avoid duplicate coding of publications. All publications were then categorized into three categories for increased utility of comparison; Articles, Reviews (Including Short Surveys), and Opinions (Includes Editorials, Letters, and Notes). 

Research assistants evaluated all downloaded publications and excluded publications that contained no ENDS content, including publications that contained our search keywords but did not focus on or discuss ENDS, after confirmation from a second research assistant. 

A content coding system was developed based on previous content analyses of ENDS news stories [23]. Where possible, publication themes, terminology, and statements were kept consistent with prior analyses for comparison. A pilot coding of 20 randomly selected ENDS articles from our sample was conducted during the adaptation of the previous coding system for the current study. After ensuring consistent coding among research assistants, a final coding instrument was developed which contained 40 variables. 

Each publication was coded for the geographic level of focus of the research/discussion presented (international, national, state-specific), publication type (e.g., research article, review, editorial), terminology (e.g., “e-cig”, “vape”, “mods”), ENDS themes (e.g., ENDS health or safety risks, use for cessation, regulatory issues, guidance for healthcare professionals), positive claims about ENDS made directly by authors (e.g., “effective for smoking cessation”, “less risky than cigarettes”, “no second hand smoke”), and negative claims about ENDS made directly by authors (e.g., “not effective for smoking cessation”, “prevents quitting or promotes dual use”, “gateway for children”). 

If a single sentence presented multiple points of view, it would be coded as both a positive and negative statement. Sentences that were agnostic and discussed the possibility of a positive or negative were not coded as either (i.e., “vaping may or may not help smokers quit”). Sentences describing comparative risk would be coded as a positive and a negative (i.e., “vaping produces fewer toxicants and carcinogens than smoking cigarettes”); this statement would be coded as a negative due to the claim of toxins and carcinogens, but a positive due to less relative risk compared to cigarettes. If a specific statement theme was made, it was counted just one time. For example, if the statement “e-cigarettes are less risky than cigarettes” was made five times throughout a publication, it would still just be coded one time. 

For the purpose of this project, national publications were defined as those focused on the United States, and not a specific state or region within the country. If the geographic focus was on the state-level, we coded the specific state. If the publication type was a research article, we coded the type of study design used (e.g., cross-sectional, randomized control trial, cohort study). If specific ENDS related outcomes were stated, we recorded which measures were reported (e.g., toxicant or carcinogen levels, cessation efficacy, economic effects). We also recorded if the publication made any mention of the following: Food and Drug Administration regulation, marketing restrictions, and flavors. 

We identified each journal’s country of publication to identify potential differences in ENDS positivity/negativity and thematic contrasts by country. This analysis was based on the observation of various ENDS position papers and public statements from organizations in several countries over the years. This assessment considered the country where the journal is published rather than the county of origin of the authors. India was dropped from our journal origination analysis because it only included a single publication, limiting the power of these comparisons.

To assess inter-rater reliability after the pilot phase, two research assistants both coded a random selection of 10% of publications, with a resulting Cohen’s Kappa statistic of 0.89, signifying very good agreement. All discrepancies were resolved during the pilot phase by re-reviewing publications and through group discussion. Descriptive statistics were used to present the frequency of publication themes and statements and Chi-square tests were conducted to compare the variable distribution by publication type as well as journal’s country of origin. To minimize likelihood of a Type 1 error from multiple tests, we used a conservative significance level of 0.01 for all analyses using R (Version 3.4.2) (R Core Team, Vienna, Austria).

## 3. Results

**Figure 1:**
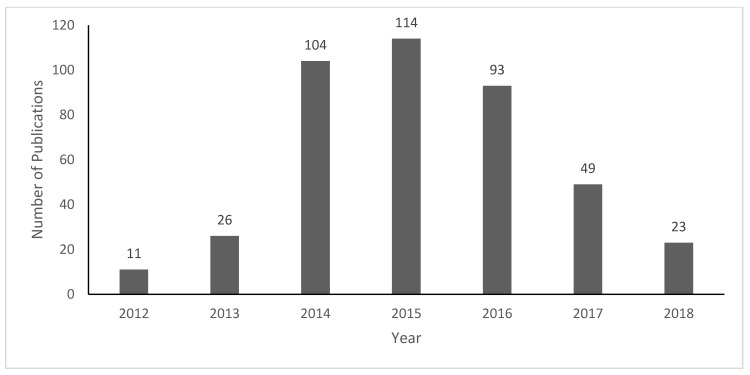
Electronic cigarette publications in core clinical journals from 2012–2018.

Searching the core clinical journals on PubMed for our keywords resulted in 555 publications. After applying inclusion and exclusion criteria, a total of 421 publications were coded and analyzed. Figure 1 shows the frequency of core clinical ENDS publications by year between 2012 and 2018. The number of publications peaked in 2015 (*n* = 114), and decreased annually since 2015, with only 23 publications in 2018. 

### 3.1. Publication Type, Study Design, and Journal

Of the 421 publications, 105 were research articles (24.9%), 100 were letters (to the editor) (23.8%), 63 were notes (15.0%), 55 were editorials (13.1%), 53 were short surveys (12.6%), and 45 were reviews (10.7%). 

Of the 105 research articles, 40 utilized a cross-sectional study design (38.1%), 18 were cohort studies (17.1%), 5 were randomized control trials (4.8%), 3 were case control studies (2.9%), and 39 used other design types (37.1%).

The top 10 core clinical journals with electronic cigarette publications were The British Medical Journal (BMJ) (n = 103, 24.5%), Pediatrics (*n* = 33, 7.9%), The Lancet (*n* = 28, 6.7%), The New England Journal of Medicine (*n* = 26, 6.2%), The American Journal of Public Health (*n* = 26, 6.2%), The Canadian Medical Association Journal (*n* = 20, 4.8%), Journal of the American Medical Association (*n* = 19, 4.5%), Journal of the American Medical Association: Pediatrics (*n* = 19, 4.5%), CHEST (*n* = 19, 4.5%), and Annals of Internal Medicine (*n* = 18, 4.3%).

### 3.2. Geographic Level of Focus

Of the 421 publications, 239 had a US national focus (56.8%), 158 had an international focus (37.6%), and 24 focused on particular US states (5.7%). Of the 24 publications that had a state-level focus, 8 were focused on California (33.3%), 3 on Connecticut (12.5%), 3 on New York (12.5%), 2 on Hawaii (8.3%), and 2 on Kentucky (8.3%). Alaska, Illinois, Michigan, Minnesota, Oklahoma, and ‘Midwest states’ were each the focus of a single study.

### 3.3. Terminology, Themes, and Outcomes

Table 1 shows terminology, publication themes, and outcomes present by the type of publication. This table referred to any statement made, regardless of whether it referenced a single device or all device combinations. “E-cig” and “electronic cigarette” were the most common terms used, being mentioned in 96.2% of all analyzed publications. The terms “Vaporizer” and “Vape” were the second most common ENDS-related terms used (32.1%), followed by “electronic nicotine delivery system” (26.7%) and “mods” and/or “tanks” (3.3%). About 8% of all publications referred to “e-juice” and/or “e-liquid”. 

The frequency of themes covered in ENDS studies was significantly associated with publication type. The most common theme in all publications was health or safety risks (64.1%), found mostly in reviews (80.6%). Policy or regulation issues were present in 53.0% of all publications, and most common in reviews (72.4%). Patterns of use and prevalence was the theme of 46.6% of all publications, and was most commonly found in articles (62.9%). The theme “use for cessation” was in 45.6% of all publications, and was found most in reviews (59.2%). Perceptions about ENDS (i.e., a study reporting on how a group felt about ENDS) was found in 21.6% of all publications, and had the highest prevalence in articles (32.4%). The least common theme, guidance for healthcare professionals, was prevalent in just 12.6% of publications. 

In terms of ENDS outcomes measured or discussed, 15.2% of all publications provided data and results about ENDS cessation efficacy and 15.0% provided data and results on ENDS toxicant levels. Among publications that provided ENDS toxicant data, 10.0% compared these levels to toxicant levels in cigarettes. Publications also discussed flavors in ENDS (37.8%), United States Food and Drug Administration (FDA) regulation (33.3%), and marketing restrictions (32.5%).

### 3.4. Positive, Negative, and Neutral Statements

Table 1 also displays the percentage of core clinical journals mentioning positive, negative, and other statements by the publication type. Regarding positive statements, 34.0% of publications mentioned that e-cigarettes are less risky than cigarettes, points made significantly more frequently in reviews (48.0%) compared to articles (28.6%). One quarter (24.9%) of all publications mentioned ENDS were or could be effective for smoking cessation, while few publications (1.9%) referred to a lack of second-hand smoke with ENDS.

Regarding negative statements, 36.3% of all publications stated ENDS appealed to children or acted as a gateway to other forms of tobacco, 30.4% mentioned ENDS exposed users to toxins or carcinogens, and 30.2% stated nicotine is addictive or harmful. Another 27.3% of all publications mentioned ENDS prevented quitting or promoted dual use of tobacco products and 27.3% stated the health effects of ENDS are unknown. We found that 17.6% of all publications mentioned ENDS were not effective for smoking cessation.

### 3.5. Positivity and Negativity Trends

**Figure 2:**
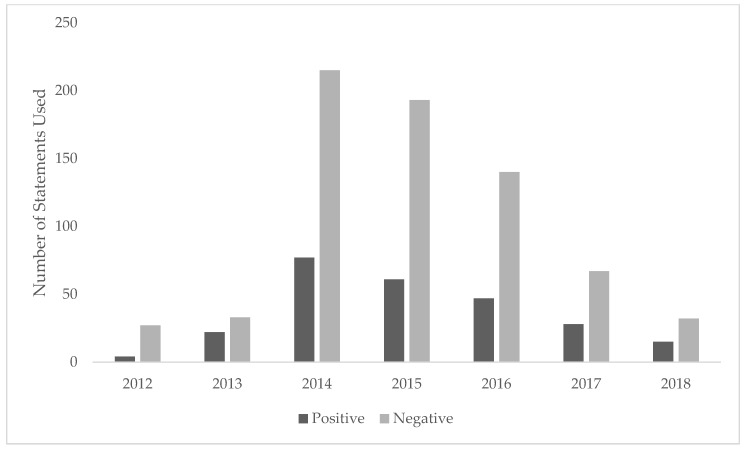
Number of positive and negative statements in electronic nicotine delivery system publications found in core clinical journals over time. Each specific statement was counted a maximum of one time per publication.

Figure 2 shows the number of unique positive and negative statements about ENDS made over time in unique publications. The number of positive and negative statements year to year roughly followed the pattern of number of publications (Figure 1). However, Figure 2 shows a substantially higher number of negative statements in all years, especially between 2014–2017. Although both positive and negative statements declined after 2014, the rate of decrease in negative statements was much higher, such that by 2018, the ratio of negative to positive statements was considerably smaller than in previous years. 

### 3.6. Journal’s Country of Publication

Over the time period of this analysis, statements from national organizations in different countries varied in terms of their general positive or negative sentiments regarding ENDS. Therefore, we chose to analyze themes based on country where the journal was published. The journals represented in our sample originated from the United States (*n* = 42 journals), the United Kingdom (*n* = 6 journals), and Canada (*n* = 1 journal). There were 260 publications from journals published in the United States, 140 publications in journals published in the United Kingdom, and 20 publications in a single Canadian Journal. 

There was a significant association between the mention of various ENDS risks/negative statements and journal country of origin. Compared to publications from US journals, publications from the UK journals less frequently referred to ENDS as having unknown health effects, as exposing individuals to toxins or carcinogens, and as containing nicotine/being addictive (Table 2).

## 4. Discussion

The number of ENDS publications in core clinical journals increased rapidly from 2012, and peaked in 2015. The number of publications grew at the same time as ENDS awareness and use increased [29,30]. The frequency of publications has declined each year since 2015. There were fewer ENDS publications in core clinical journals in 2018 compared to 2013. A recent analysis of all ENDS publications found that 2018 had the highest number of publications to date [31]. In contrast, our analysis restricted to core clinical journals found a low number of publications in 2018, potentially indicating that clinical journals are focusing on other topics or authors are choosing to publish in non-core clinical journals. Additionally, this might correspond to the Food and Drug Administration (FDA) Deeming Rule in 2016 which gave the FDA the authority to regulate ENDS; the FDA’s Center for Tobacco Products funds research projects that might shift prioritization from clinical research on ENDS to other areas, such as regulation or marketing.

In terms of article type, most reviews discussed the use of ENDS for cessation purposes, but only a small percentage (18.4%) of reviews provided guidance for healthcare professionals. This was surprising because the use of ENDS for smoking cessation, either positively or negatively, is a widely debated topic in public health. We found core clinical reviews to be more positive and negative compared to core clinical articles. One potential hypothesis is that reviews are referencing non-clinical articles which could support more positive and negative findings. 

When examined over time, the number of negative statements about ENDS were much higher than positive statements, however the decline in number of negative statements has outpaced those of positive ones since 2014. In particular, negative statements such as unknown health effects, toxins, and carcinogen exposure decreased over time. Concerns regarding the gateway effect and appeal to children increased from 2012, indicating a potential shift in focus. It should be noted that these analyses occurred prior to the recent attention to e-cigarette or vaping-device associated lung injury (EVALI) in 2019 [32]. Future research should include 2019 data to observe how EVALI is discussed in the literature. 

When we compared positive and negative statements made in journals based upon country of journal origin, we found that journals in the US had a higher percentage of negative statements regarding unknown health effects, nicotine harms, and exposure to toxins or carcinogens compared to those from the UK. The United Kingdom has been on the forefront of the harm reduction debate [33], and these attitudes might potentially be captured in the country’s journals. These findings may have been different if we analyzed by author affiliation, but the findings remain interesting given we found differences in positivity/negativity in the way we would hypothesize. These findings should be interpreted with caution, but the results reinforce the idea that there is a geographic component that influences beliefs and perceptions of ENDS that could impact clinical practice. Overall, we think the findings from this study will be useful to understand the electronic cigarette literature the physicians are being exposed to. As we mentioned in the introduction, this is important because a majority of physicians in the US report changing clinical practice due to reading literature. A strength of this study is that it builds upon a recent content analysis of electronic cigarette news stories [23], incorporating the same risk and benefit statements. This allows for increased future comparison between the different media sources. 

One limitation of this study is the restriction to core clinical publications which may miss journals physicians routinely access, however, this appears to be the best group of literature that physicians commonly request. Another potential limitation is our decision to present counts of positive and negative statements over time. This decision could skew results as longer manuscripts can contain more statements, but this is a more objective measure than deciding if an overall paper is positive, negative, or leaning in one direction. Another limitation is our decision to analyze by the country of the journal rather than the country of the authors; future studies would benefit from looking at specific author affiliations. Additionally, we did not distinguish whether positive or negative statements were made towards specific ENDS devices, but our analysis found that nearly all publications made general references to “e-cigarettes” or “ENDS devices.” 

## 5. Conclusions

As more evidence emerges regarding the health effects of ENDS, messages that clinicians relay to their patients might change over time. Physicians are an important and trusted source of information for their patients, and patients considering switching to e-cigarettes will expect their physicians to be knowledgeable about ENDS health effects and effectiveness for smoking cessation. Future studies should explore how the publication contents differ when the focus of a publication is on adults, adolescents, or pediatrics. Future studies should analyze the contents of ENDS publications beyond the core clinical journals so we have a larger picture of the content physicians and researchers are being exposed to. Future studies should also compare the content in journals to other sources of media and information that can influence a physician’s attitude towards ENDS. 

## Figures and Tables

**Table 1:** Prevalence and trends of electronic nicotine delivery system publications in core clinical journals using specific terms, themes, belief statements, and outcomes.

Publication Type:	Article (%) (*n* = 105)	Reviews (Includes Short Survey) (%) (*n* = 98)	Opinions (Letters, Notes, Editorials) (%) (*n* = 218)	Total—All Publications (%) (*n* = 421)	*p*-Value ^2^
**Terminology Present**					
E-Cig, Electronic Cigarette	95.2	98.0	95.9	96.2	0.569
Vaporizer, Vape	28.6	49.0	26.2	32.1	<0.01
ENDS ^1^	26.7	23.7	16.1	20.4	0.058
E-Juice, E-Liquid	10.5	12.4	4.6	7.8	0.031
E-Hookah	3.8	5.1	2.8	3.6	0.783
Mods, Tanks	3.8	9.2	0.5	3.3	<0.01
JUUL	0.0	2.1	0.5	0.7	0.179
**Themes Present**					
Health or safety issues	57.1	80.6	60.1	64.1	<0.01
Policy or regulation issues	41.0	72.4	50.0	53.0	<0.01
Prevalence	62.9	51.6	36.7	46.6	<0.01
Use for cessation	34.3	59.2	45.0	45.6	<0.01
Perceptions about e-cigs	32.4	22.7	16.1	21.6	<0.01
Guidance for healthcare professionals	8.6	18.4	11.9	12.6	0.149
**Positive Statements Mentioned**					
Less Risky than Cigarettes	28.6	48.0	30.3	34.0	<0.01
Effective for Cessation	21.9	34.7	22.0	24.9	0.055
No Secondhand Smoke	0.0	4.1	1.8	1.9	0.100
**Negative Statements Mentioned**					
Gateway, Appeals to Children	30.5	46.9	34.4	36.3	0.046
Toxin, Carcinogen Exposure	32.4	39.8	25.2	30.4	0.039
Nicotine is Addictive, Harmful	28.6	37.8	27.5	30.2	0.215
Prevents Quitting, Promotes Dual Use	41.0	31.6	18.8	27.3	<0.01
Health Effects Unknown	36.2	27.6	22.9	27.3	0.043
Not Effective for Cessation	21.0	22.7	13.8	17.6	0.093
**Article Discusses**					
Flavors	36.2	57.1	29.8	37.8	<0.01
FDA Regulation	39.1	47.0	24.3	33.3	<0.01
Marketing Restrictions	23.8	52.0	28.0	32.5	<0.01
**Outcomes Present**					
Cessation Efficacy	13.3	23.7	12.4	15.2	0.029
Toxicant Levels	15.2	20.4	12.4	15.0	0.248
Compared to Cigarettes	7.6	13.3	9.6	10.0	0.522

^1^ Electronic Nicotine Delivery System. ^2^ Differences by Publication Type assessed using Chi-square test.

**Table 2:** Percentage of electronic nicotine delivery system publications with specific belief statements and characteristics, stratified by core clinical journal’s country of origin.

Characteristics	Journal’s Country of Origination			
	United States	United Kingdom	Canada	*p*-Value ^1^
Number of Publications	260	140	20	
Number of Journals	42	6	1	
**Positive Statements Mentioned**				
Less Risky than Cigarettes	32.3%	38.6%	20.0%	0.175
Effective for Cessation	22.3%	30.7%	20.0%	0.139
**Negative Statements Mentioned**				
Toxin, Carcinogen Exposure	38.1%	17.9%	15.0%	<0.01
Nicotine is Addictive, Harmful	35.8%	22.9%	10.0%	<0.01
Health Effects Unknown	32.7%	15.7%	35.0%	<0.01
Prevents Quitting, Promotes Dual Use	30.4%	21.4%	30.0%	0.170
Not Effective for Cessation	21.5%	11.4%	10.0%	0.026

^1^ Difference by Journal Country of Origin assessed using Chi-square test.
